# Liquid Embolic Agents in Spectral X-Ray Photon-Counting Computed Tomography using Tantalum K-Edge Imaging

**DOI:** 10.1038/s41598-019-41737-6

**Published:** 2019-03-27

**Authors:** Isabelle Riederer, Daniel Bar-Ness, Melanie A. Kimm, Salim Si-Mohamed, Peter B. Noël, Ernst J. Rummeny, Philippe Douek, Daniela Pfeiffer

**Affiliations:** 1 Department of Diagnostic and Interventional Radiology, Technical University of Munich, School of Medicine, Munich, Germany; 2 Department of Diagnostic and Interventional Neuroradiology, Technical University of Munich, School of Medicine, Munich, Germany; 3 University Claude Bernard Lyon 1, CREATIS, CNRS UMR 5220, INSERM U1206, INSA-Lyon, France; 4 Department of Interventional Radiology and Cardio-vascular and Thoracic Diagnostic Imaging, Louis Pradel University Hospital, Bron, France

## Abstract

The aim was to evaluate the potential of Spectral Photon-Counting Computed Tomography (SPCCT) to differentiate between liquid embolic agents and iodinated contrast medium by using tantalum-characteristic K-edge imaging. Tubes with a concentration series of tantalum and inserts with different concentrations of iodine were scanned with a preclinical SPCCT system. Tantalum density maps (TDM) and iodine density maps (IDM) were generated from a SPCCT acquisition. Furthermore, region-of-interest (ROI) analysis was performed within the tubes in the conventional CT, the TDM and IDM. TDM and IDM enable clear differentiation between both substances. Quantitative measurements of different tantalum concentrations match well with those of actually diluted mixtures. SPCCT allows for differentiation between tantalum and iodine and may enable for an improved follow-up diagnosis in patients after vascular occlusion therapy.

## Introduction

Arteriovenous malformation (AVM) is a vascular anomaly with a connection between arteries and veins and a lack of intervening capillary bed. AVMs can occur in the central nervous system with a prevalence of <1%^1–4^. Depending on multiple factors such as localization, size, feeding arteries and draining veins, AVMs can be treated by endovascular embolization, surgery, radiosurgery or by their combinations^5–7^. A variety of embolic agents has been introduced^6,8,9^ that can be divided into solid and liquid agents, with the latter being more commonly used. One of the essential requirements for these glues is  radiopacity to control the process of intervention, success of vessel occlusion or potential complications in digital subtraction angiography.

Current liquid embolic agents are Onyx (producer: Covidien, eV3 Neurovascular, Irvine, Calif. USA)^10–12^ and Squid (producer: emboflu, Gland, Switzerland)^13^ consisting of ethylene vinyl alcohol copolymer dissolved in dimethyl sulfoxide (DMSO). Micronized tantalum powder is added for radiopaque visualization.

**Figure 1:**
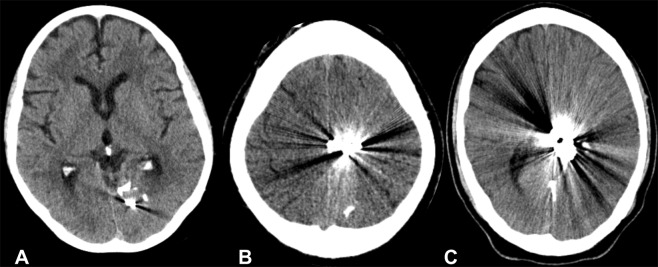
Un-enhanced CT scans of the brain after embolization of Arteriovenous malformations (AVMs) with Onyx, in the left occipital lobe (**A**), in the left parietal region (**B**) and left parietotemporal region (**C**). Noticeable are the artifacts that reduce diagnostic image quality.

CT-angiography of the brain after embolization can be necessary to assess possible remaining feeders as well as the nidus in order to plan further procedures. The micronized tantalum powder, added to the liquid embolic agent for  radiopacity, however, can cause severe beam hardening artifacts in these CT images. Sometimes it is difficult to appropriately assess brain tissue and vessels around the clot of liquid embolic agent (Fig. 1). This might affect diagnosis and therapy management^14–16^. In these cases, techniques reducing artefacts would be desirable to improve image quality.

Advanced CT imaging methods have been developed in the last years providing spectral information^17^ for the analysis of tissue composition as X-ray attenuation is energy- and material-dependent. Recently, several Spectral Photon-Counting CT (SPCCT) systems have been introduced, where X-ray photons are individually counted and spectrally binned by analyzing the pulse heights generated in a semi-conductor detection layer^18–23^. This concept allows to incorporate a multiple (more than two) energy bins for energy-selective data acquisition. Using one such prototype system, we could show that the SPCCT system enables differentiation between gadolinium-based and non-ionic iodine-based contrast material in a colon^24^ and liver^25^ phantom. Furthermore, it has been demonstrated that discrimination between gold nanoparticles and iodinated contrast agent is possible in different organs *in vivo* in animals using the SPCCT system^26^. First experiences of human imaging using a SPCCT system were carried out in cadaver^18^ and phantom studies^27^ or *in vivo* regarding the abdominal system^28^ and the vascular system of the head and neck^29^.

The aim of this specific study was to explore the feasibility of SPCCT for material decomposition of tantalum using its characteristic K-edge. Furthermore, we intended to analyze the potential of SPCCT for the differentiation between tantalum and iodine for an improved visualization.

## Results

**Figure 2:**
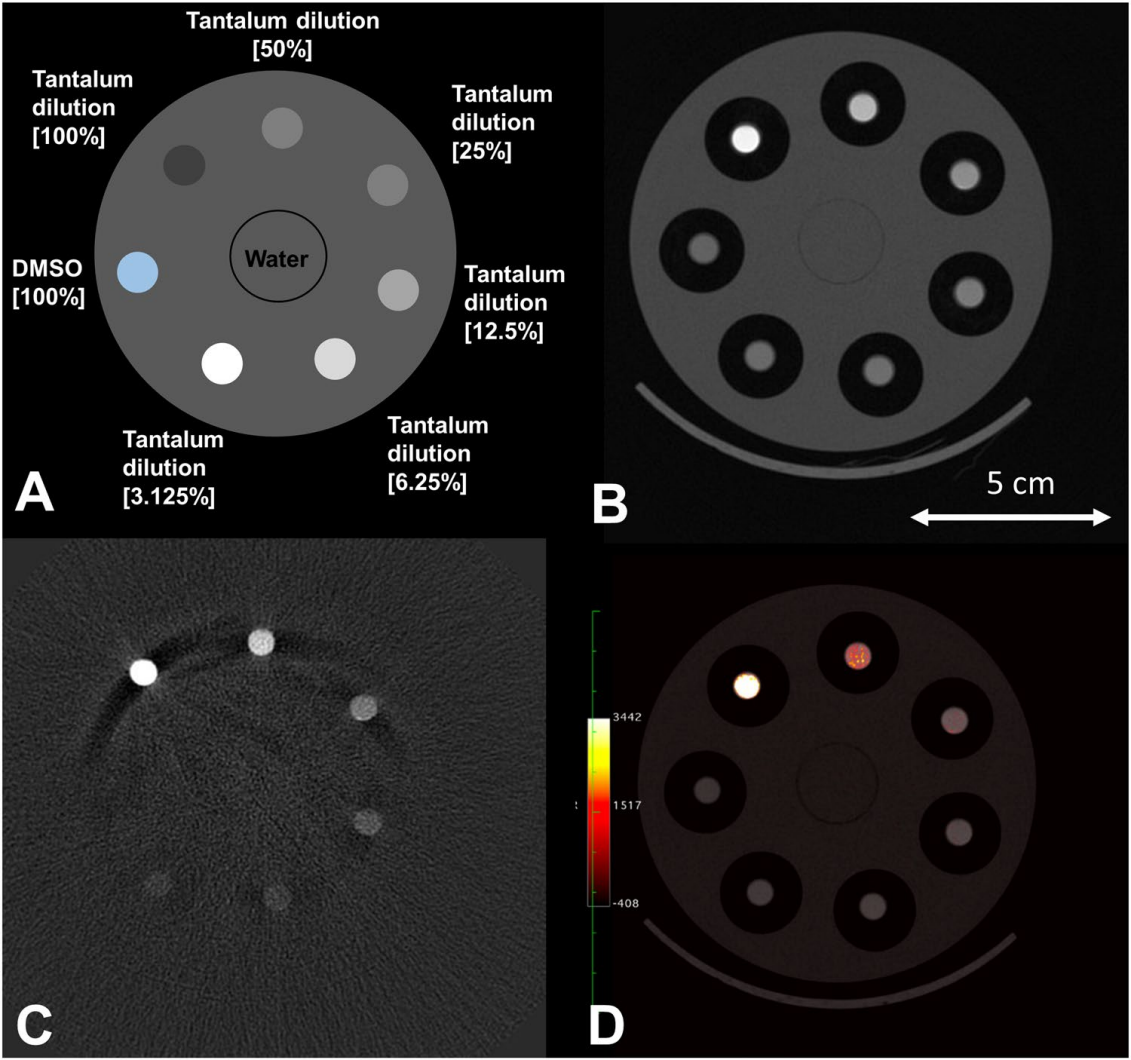
CT-Scan of the phantom model containing tubes with a concentration series of tantalum diluted in DMSO. (**A**) Schematic of the phantoms contents, (**B**) HU image, (**C**) tantalum density map, and (**D**) fusion image of (B + C) using OsiriX (http://www.osirix-viewer.com).

Figure 2 displays the results of the material decomposition for tantalum that is provided by the SPCCT system. Figure 2A shows a schematic explaining the content of the different tubes with a dilution series of tantalum in the range from 3.125% to 100% and a control tube containing DMSO. A conventional CT image of the phantom is contained in Fig. 2B, where one can observe that it is difficult to discriminate between the different dilutions in the low ranges. In the obtained tantalum density maps (TDM) (Fig. 2C**)**, however, already the smallest dilution could be visually discriminated from the control tube containing pure DMSO (100%). Figure 2D contains an overlay of the conventional HU image and the TDM.

**Figure 3:**
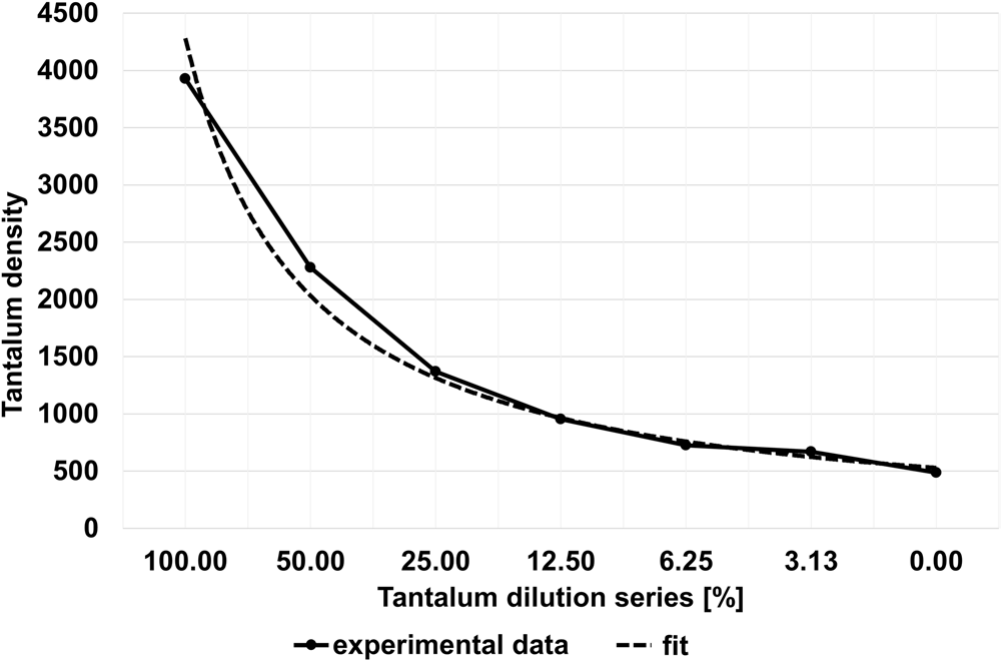
Calibration curve of tantalum showing correlation between tantalum density values and the prepared tantalum dilution series. 100% tantalum corresponds to a mixture of 1/3 Squid18 and 2/3 DMSO.

**Figure 4:**
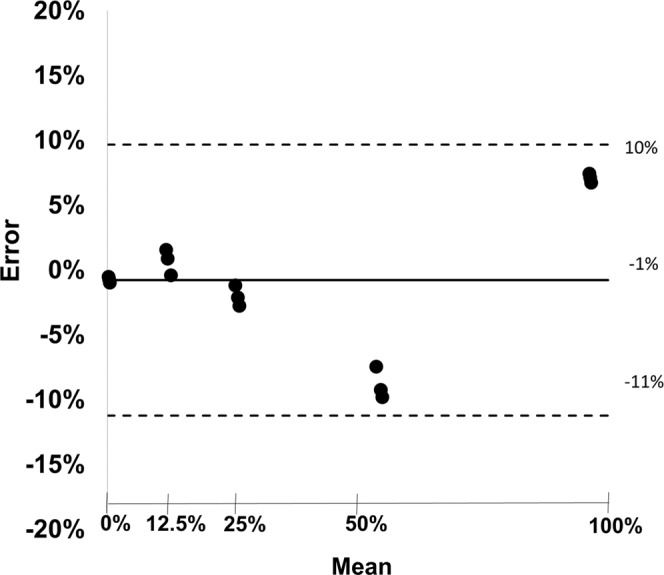
Bland-Altman plot showing difference between measured and expected tantalum concentrations versus average of true and expected tantalum concentrations. The black line represents the bias and the dashed lines represent upper and lower limits of the mean (confidence limits ± 1.96).

Figure 3 shows a calibration curve of the tantalum material. With its aid, additional scans were analyzed and tantalum dilution concentrations were calculated. The quantitative measurements of the tubes with different tantalum concentrations matched well with the actually known mixtures (measured: 92.92 ± 0.004%, expected: 100%; measured: 58.85 ± 0.01%, expected: 50%; measured: 27.03 ± 0.01%, expected: 25%; measured: 11.84 ± 0.01%, expected 12.5%; measured: 0.73 ± 0.002%, expected: 0%; RMSE = 0.05). A corresponding Bland-Altman plot is displayed in Fig. 4, highlighting the difference between measured and expected tantalum concentrations versus the average of measured and expected tantalum concentrations. All measurements are located within the range of confidence limits (1.96).

**Figure 5:**
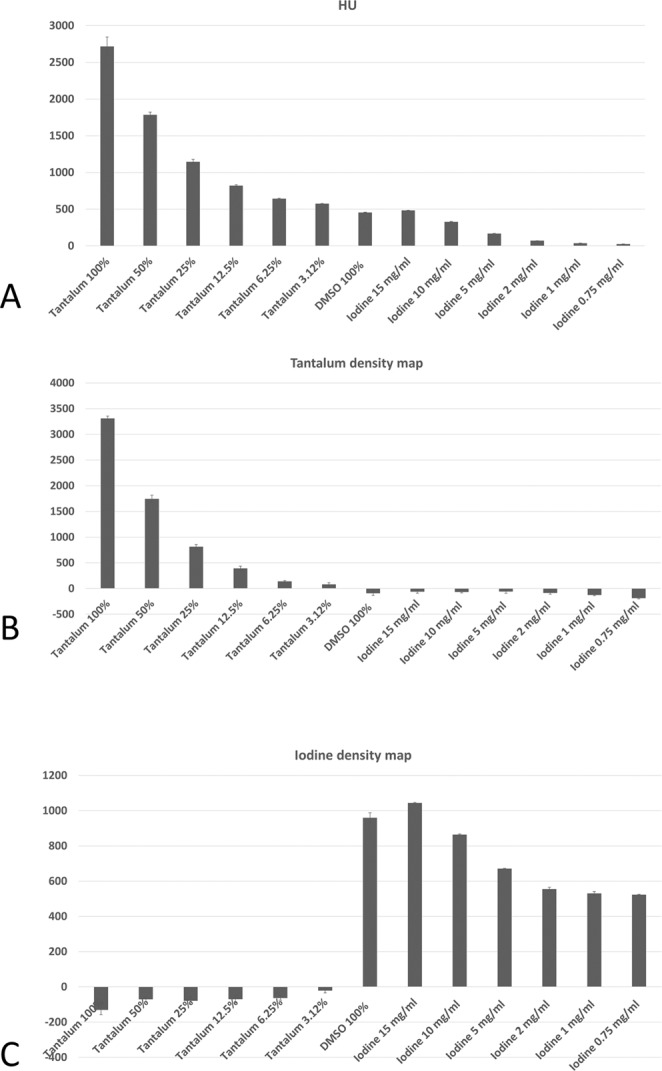
Bar plots showing the values of the region-of-interest (ROI) analyses within the tubes in Hounsfield Units (HU), tantalum density maps and iodine density maps.

We observe that the TDMs and IDMs provided by SPCCT enable clear differentiation between tantalum and iodine. This is shown in Fig. 5, where Fig. 5A represents the conventional HU values, Fig. 5B the TDM and Fig. 5C the IDM. In the conventional HU-CT, the values of the tubes containing low concentrated tantalum, DMSO or high concentrated iodine are similar and can, therefore, not be differentiated. In the TDM, the values of the tubes correspond to the prepared dilution series; the tubes containing DMSO and iodine have values below 0. In the IDM, the dilution series of iodine can be reproduced and tantalum has negative values. DMSO has similar high values as high concentrated iodine, obviously due to similar physical background.

## Discussion

In this study, we demonstrated that SPCCT allows for material decomposition of tantalum and discrimination between tantalum and iodine.

Recently, we could already show that spectral images using different techniques allow for material quantification and that reliable measurements of iodine concentrations are possible even for very low concentrations of 0.5 mg/ml^30,31^. As SPCCT offers the potential to improve image quality and to lower image noise, further studies have to be performed using this promising novel technique.

Photon-counting detectors provide the possibility to measure the energy level of each detected photon based on pulse height analysis and enables material-specific imaging in CT by delivering information about energy-based attenuation profiles of tissues^20^. One highlight of the SPCCT system is its ability to specifically detect exogenous contrast media due to edges in the X-ray attenuation profiles of elements such as gold^32^. Since tantalum has its K-edge binding energy in the relevant energy range of the X-ray spectrum (67.4 keV), K-edge imaging of tantalum is feasible in the clinical setting. One study has recently been published using K-edge imaging of the SPCCT system^33^ to differentiate between two contrast agents (iodine and gadolinium) *in vivo*. The advantage of using SPCCT for tantalum imaging has been highlighted in our study. Material decomposition of tantalum is possible enabling discrimination between iodinated contrast agent and liquid embolic agent – containing tantalum powder. One study could show that tantalum has high attenuation, generates high contrast and provides higher signal and better element-specific image CNR in SPCCT over tungsten, gold and bismuth^34^. Furthermore, another study could show that tantalum enables to reduce the amount of contrast medium and radiation dose due to higher contrast enhancement and greater contrast-to-noise ratio compared to iodine-based contrast agent, and, thus, may improve vascular imaging in overweight patients^35^. A recent published study^29^ concluded that photon-counting CT might improve image quality of CT angiography compared to conventional single-energy CT scans using energy-integrating detectors CT. To summarize, it is obvious that photon-counting CT might improve image quality of CT scans of patients after AVM embolization.

First results of X-ray photon-counting CT scans of the brain *in vivo* could show a greater gray-white matter contrast compared with conventional CT^36^. CT scans of the brain are common in the clinical routine while evaluating brain injury in emergency cases or for follow-up controls. Beam hardening artifacts especially near to the base of the skull might affect diagnosis and potentially mimic intracranial hemorrhage. Also here, Photon-Counting CTs might be a promising technique to improve image quality.

One study^18^ used a photon-counting-detector CT scanner that is capable to image human-sized objects. The rotation time was 1.0 or 0.5 seconds, and is close to a conventional CT scanner with about 0.4 sec.^37^. Thus, it seems applicable for clinical use in the future.

One of the major concerns about CT technique is the radiation exposure with possible risks of cancer or cataract. One study concluded that photon-counting CT imaging is possible at clinical dose rates with clinical levels of image quality, and furthermore, improved CNR relative to state-of-the-art CT^18^. A review about photon-counting CT^38^ concludes that photon-counting CT can minimize image noise and increase spatial resolution that will enable to reduce radiation doses up to 30–40%.

In this phantom study we used a tube current of 100 mA and a tube voltage of 120 kVp that is comparable to another study scanning human heads *in vivo* with a clinical spectral dual-layer CT scanner (120 kVp and 260 mAs^39^).

The potential advantages of the SPCCT system, in particular material decomposition and artifact reduction, might also facilitate interpretation of CT examinations of patients after embolization therapies of brain AVMs. A frequent reported disadvantage of Onyx is the production of artifacts that often makes it difficult to interpret the areas near to the embolization material whether there is hemorrhage, the most important complication^40^, or a remaining feeder. Furthermore, the artifacts caused by the liquid embolic agents might affect planning of a subsequent radiotherapy and might lead to higher radiation doses^16^. Magnetic resonance imaging (MRI) instead of CT imaging might be a way to solve this challenge, but may not always be available or not possible in cases with contraindications such as a cardiac pacemaker. Furthermore, but to a lesser extent, Onyx can also cause susceptibility artifacts in MRI^16^.

Another solution to reduce artifacts due to the liquid embolic agents is the use of new embolic agents; one study could show that the precipitating hydrophobic injectable liquid “PHIL” produces fewer artifacts than Onyx in an *in vivo* model by using covalently bound iodine instead of tantalum powder for ^radiopacity41,42^. However, these agents are relatively new and further studies are required to evaluate their efficacy. The results of our study *in vitro* are promising to reduce artifacts caused by tantalum and to improve image quality in patients after AVM embolization. Further studies *in vivo* are necessary to assess the potential impact of the SPCCT system.

A limitation of this study is that the absolute content of tantalum of the used liquid embolic agent (Squid 18) is unknown. We used a concentration series for our experiments. Additionally, DMSO has a high signal due to its chemical structure. In patients, DMSO is injected into the tubes before the injection of the liquid embolic agent to minimize the risk that it becomes hard within the tubes. Within the brain of the patient, DMSO is diluted and diffuses into the tissue, so this problem does not occur with patients. Furthermore, the errors in quantitative measurements were larger when measuring higher amounts of tantalum. One explanation could be that higher concentrations of tantalum lead to higher attenuation and thus photon starvation, which results in more artifacts resulting in higher errors. Further studies have to be performed to evaluate this observation.

To conclude, Spectral Photon-Counting CT provides tantalum density maps and allows for material decomposition and differentiation between tantalum and iodine *in vitro*. Therefore, the introduction of SPCCT into the clinical field may improve diagnostic imaging especially in patients after embolization of AVMs.

## Materials and Methods

### Scan specimens

We used Squid^13^ the as a tantalum-based liquid embolic agent for endovascular occlusion as it is used in human patients. We diluted the stock solution Squid18 (emboflu, Gland, Switzerland) with DMSO (1/3 Squid18 + 2/3 DMSO) to gain a working dilution suspension and performed a serial dilution as follows: 100%; 50%; 25%; 12.5%, 6.25%; 3.125%. Additionally, a tube was filled with 100% DMSO as control reference.

Inserts with different concentrations of iodine (0.75 mg/ml; 1 mg/ml; 2 mg/ml; 5 mg/ml, 10 mg/ml and 15 mg/ml; QRM GmbH, Möhrendorf, Germany) were used. All tubes and inserts were embedded into a solid cylinder of water-equivalent material and 10 cm diameter. Each scan included three different scan positions and eight slices.

### Spectral Photon-Counting CT

All experiments were performed with a five bin X-ray spectral photon-counting computed tomography (SPCCT) system (Philips Healthcare, Haifa, Israel) derived from a modified clinical CT system to obtain spectral and conventional data^21^. This system is provided with energy-sensitive photon-counting detectors made of the direct conversion high band gap semiconductor cadmium zinc telluride. The in-plane field of view was 168 mm, and the z-coverage of the scanner at the isocenter was 2.5 mm. Axial scans over 360° were obtained with a tube current of 100 mA, a tube voltage of 120 kVp, and a scanner rotation time of 1 second.

### Material decomposition and quantitative measurements

Multi-bin photon-counting data were pre-processed, and a conventional CT image was derived from the summed information contained in all energy bins. In addition, after pileup correction, the multi-bin counting data were used to perform a maximum likelihood-based material decomposition into a water and iodine material basis^19,20^ in projection space. The material-decomposed projections have been reconstructed using FBP with a standard filter kernel and no post processing was done to further reduce image noise on FBP images. The iodine and virtual non-contrast images were averaged to a slice thickness of 1 mm after CT reconstruction.

### Region of interest analysis

First, a reference scan with the dilution series of tantalum was performed to calibrate the following measurements. Then, measurements of all tubes and inserts, as described above, were performed. Region-of-interest (ROI) analysis was performed within the tubes in the conventional images, the TDM and IDM using an image processing program (ImageJ, National Institutes of Health (NIH), United States^43^). Circular ROIs were set in the center of the tubes with a volume of 10 mm².

### Statistical analysis

Bland-Altman analysis was performed to determine the agreement between measured and expected tantalum concentrations, additionally root mean square error (RMSE) was calculated.

## Data Availability

The datasets analyzed during the current study are available from the corresponding author on reasonable request.
